# Effect of Early Initiation of Evolocumab on Lipoprotein(a) in Patients with Acute Myocardial Infarction: Sub-Analysis of a Randomized Controlled Trial

**DOI:** 10.3390/jcdd9050153

**Published:** 2022-05-12

**Authors:** Tomoaki Okada, Toru Miyoshi, Masayuki Doi, Kazumasa Nosaka, Ryu Tsushima, Satoko Ugawa, Wataru Takagi, Masahiro Sogo, Masahiko Takahashi, Hiroshi Ito

**Affiliations:** 1 Department of Cardiology, Kagawa Prefectural Central Hospital, 1-2-1 Asahi-machi, Takamatsu 760-8557, Japan; ju_10monomoto0622@yahoo.co.jp (T.O.); mdoimd@gmail.com (M.D.); kn_orch@yahoo.co.jp (K.N.); shimaryu0910@gmail.com (R.T.); satokougauga@hotmail.com (S.U.); t_wataru1206@yahoo.co.jp (W.T.); msogo0517@gmail.com (M.S.); masahiko0707@infoseek.jp (M.T.); 2 Department of Cardiovascular Medicine, Faculty of Medicine, Dentistry and Pharmaceutical Sciences, Okayama University, 2-5-1 Shikata-cho, Okayama 700-8558, Japan; itomd@md.okayama-u.ac.jp

**Keywords:** evolocumab, pitavastatin, lipoprotein(a), percutaneous coronary intervention, hypolipidemic agents, myocardial infarction

## Abstract

Elevated circulating lipoprotein(a) levels are associated with an increased risk of cardiovascular events. We reported that early initiation of evolocumab, a proprotein convertase subtilisin/kexin type 9 inhibitor, in addition to a statin substantially reduced the lipoprotein(a) levels in patients with acute myocardial infarction (AMI) after primary percutaneous coronary intervention (PCI). This sub-analysis sought to investigate the effect of evolocumab on lipoprotein(a) based on baseline lipoprotein(a) levels and characteristics. This study was a prespecified analysis of a randomized controlled trial that enrolled 102 patients who underwent primary PCI for AMI. Patients received pitavastatin (2 mg/day) alone or pitavastatin and evolocumab 140 mg subcutaneously within 24 h and 2 weeks after the index PCI. The evolocumab group showed significantly suppressed lipoprotein(a) levels in patients with baseline lipoprotein(a) levels of ≤10 mg/dL, 10 < lipoprotein(a) ≤ 20 mg/dL, and >20 mg/dL compared with the control group, as well as similar reductions in lipoprotein(a) levels in all patient subgroups. Among these subgroups, evolocumab tended to show more favorable effects in patients with diabetes mellitus. In AMI patients, early initiation of evolocumab therapy within 24 h of primary PCI suppressed the increase in lipoprotein(a) levels within 4 weeks, regardless of baseline levels and characteristics.

## 1. Introduction

Lipoprotein(a) is a low-density lipoprotein-like particle produced by the covalent bonding of apolipoprotein(a) to apolipoprotein B-100 [1]. Circulating lipoprotein(a) levels vary widely across individuals and ethnic subgroups, mediated in large part by genetic variations at the *LPA* gene locus [2]. A meta-analysis of 126,634 participants in 36 prospective studies found that lipoprotein(a) is an independent risk factor for coronary heart disease and stroke [3]. Mendelian randomization studies have linked genetic variations at the *LPA* locus to both circulating lipoprotein(a) levels and the risk of cardiovascular disease (CVD), supporting a causal role of lipoprotein(a) in CVD pathogenesis [4,5]. Generic methods to modulate circulating lipoprotein(a) levels in daily practice are yet to be determined, and further accumulation of evidence based on monitoring of patients with high lipoprotein(a) levels (even when undergoing treatment with lipid-lowering drugs such as statins) is crucial [2,6].

Low-density lipoprotein cholesterol (LDL-C)-lowering therapy is an optimal pharmacological treatment for patients with CVDs [7,8]. However, achieving the target goal using statin alone is often difficult [8]. Additional intensive LDL-C-lowering therapy involving ezetimibe and/or a proprotein convertase subtilisin/kexin type 9 (PCSK9) inhibitor can further improve the prognosis of patients with CVD undergoing statin therapy [9,10]. PCSK9 inhibitors, such as evolocumab and alirocumab, constitute a new class of drugs that can rapidly and effectively decrease LDL-C levels in patients without atherosclerotic CVD events [11,12], but with stable ischemic heart disease [10,13]. We recently reported the efficacy and safety of early initiation of evolocumab therapy combined with a statin for patients who experienced acute myocardial infarction (AMI) and underwent primary percutaneous coronary intervention (PCI) within 24 h of symptom onset [14].

In this sub-analysis, we aimed to investigate the effect of evolocumab on lipoprotein(a) levels based on the baseline lipoprotein(a) levels and characteristics.

## 2. Results

### 2.1. Baseline Characteristics

Between October 2017 and December 2019, 102 Japanese patients were enrolled and randomly assigned to an evolocumab (*n* = 52) or control (*n* = 50) group: 89 patients (87%) had STEMI and 13 (13%) had non-STEMI. Primary PCI was performed for all patients. The baseline characteristics of patients in the two groups were well-balanced (Table 1). The mean age was 65 ± 14 years, and 11.7% of patients were women. We found that 5.8% of patients had a history of myocardial infarction, 2.9% had previous ischemic stroke, and 1% had peripheral artery disease, and 76 patients (74%) were statin-naïve.

**Figure 1:**
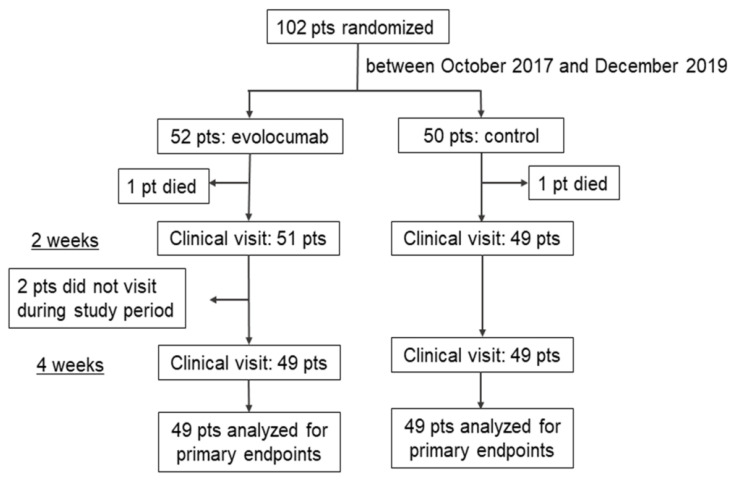
Flow diagram of this study. Patients (*n* = 102) were randomly assigned to the evolocumab group (*n* = 52) or control group (*n* = 50). After randomization, four patients were excluded, leaving a total of 98 patients for the primary outcome analysis.

The flow diagram is shown in Figure 1. After randomization, two patients (one patient in each group) were excluded from the primary outcome analyses because of death due to severe heart failure during the acute phase. Furthermore, two patients from the evolocumab group did not undergo the follow-up. Finally, 98 patients (evolocumab group, *n* = 49; control group, *n* = 49) were analyzed for the primary outcome. Changes in total cholesterol, LDL-C, triglyceride, high-density lipoprotein cholesterol (HDL-C), non-HDL-C, apoprotein B, apoprotein C3, apoprotein A1, and small dense LDL are shown in Supplementary Table S1.

### 2.2. Lipoprotein(a)

**Figure 2:**
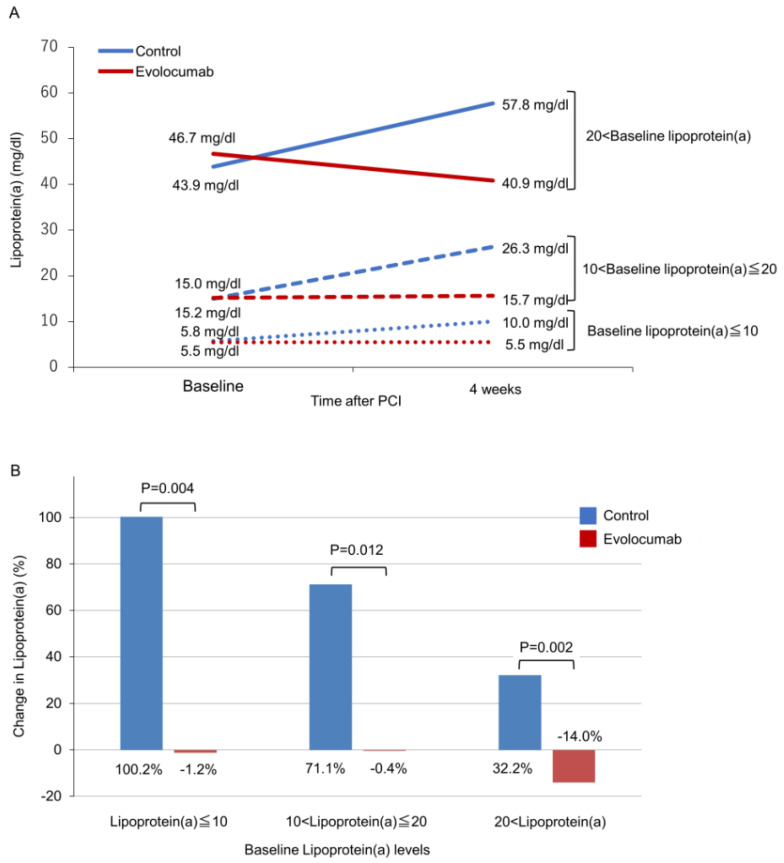
Changes in lipoprotein(a) levels. Change in mean values (**A**) and percent change (**B**) in lipoprotein(a) are shown for both groups at baseline lipoprotein(a) levels. At 4 weeks, the use of evolocumab 120 mg in addition to a statin suppressed an increase in lipoprotein(a) levels in each group compared with statin alone.

**Figure 3:**
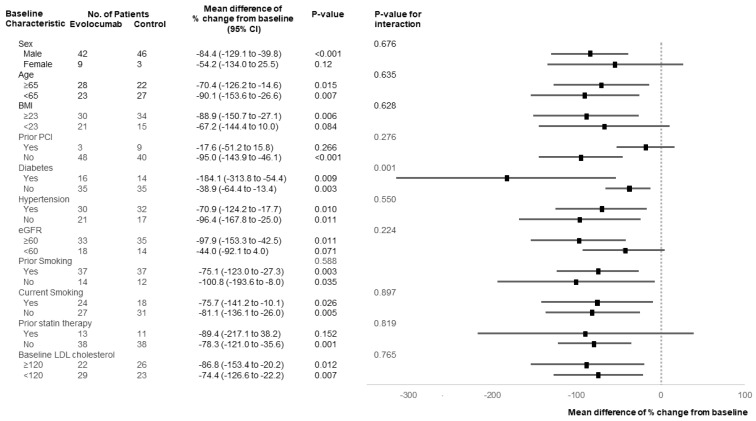
Subgroup analysis of changes in lipoprotein(a) levels. Mean difference of percent change in lipoprotein(a) levels from the baseline in subgroups is shown as a forest plot. In many subgroups, the evolocumab group tended to suppress the increase in lipoprotein(a) levels.

The change in lipoprotein(a) levels from baseline to 4 weeks was −2.7% ± 48.6% in the evolocumab group and 82.0% ± 135.9% in the control group (mean difference: −86.3%; 95% confidence interval (CI): −134.5% to −38.0%; *p* = 0.01). Next, we separately evaluated lipoprotein(a) levels in three groups based on the baseline value as follows: lipoprotein(a) ≤ 10 mg/dL, 10 < lipoprotein(a) ≤ 20 mg/dL, and lipoprotein(a) > 20 mg/dL, with reference to the baseline level, and evolocumab suppressed the increase in lipoprotein(a) levels in each group (Table 2 and Figure 2). In all groups based on the baseline value, the evolocumab group significantly suppressed the increase in lipoprotein(a) levels. Furthermore, as shown in Figure 3, the effect of evolocumab vs. control on lipoprotein(a) levels observed in the overall population at 4 weeks was similar to that in the various patient subgroups. All *p*-values for interactions, except for diabetes, were >0.05. Table 3 shows the mean difference in change in lipoprotein(a) levels between the evolocumab and control groups in patients with diabetes and without diabetes. The increase in lipoprotein(a) levels was significantly suppressed in both patients with diabetes (mean difference: −184.1%) and without (mean difference: −38.9%), whereas the interaction between patients with and without diabetes was significant (*p* = 0.001).

## 3. Discussion

We assessed the effect of early initiation of evolocumab therapy on lipoprotein(a) levels after primary PCI in patients with AMI. Evolocumab combined with pitavastatin substantially mitigated the increase in lipoprotein(a) levels, regardless of baseline lipoprotein (a) levels and patients’ characteristics. Among these subgroups, evolocumab was likely to show more favorable effects in patients with diabetes mellitus.

Previous studies have shown the effect of evolocumab on lipoprotein(a) levels in the acute phase of AMI [14,15,16] and prior PCI [17], whereas the difference in its effect among baseline patients’ characteristics has not been evaluated. For example, alirocumab, another PCSK9 inhibitor, has been reported to reduce lipoprotein(a) levels independently of the baseline kidney function [18]. However, the relevance of lipoprotein(a) lowering by PCSK9 inhibitors in patients with chronic kidney disease has yet to be established. Our study showed that evolocumab combined with pitavastatin substantially mitigated the increase in lipoprotein(a) levels, regardless of patients’ characteristics. In addition, among these subgroups, evolocumab was likely to show more favorable effects in patients with diabetes mellitus. However, this study was a post hoc analysis of a randomized, controlled trial. The sample size in each subgroup was small. Further research will be needed to confirm our findings.

In a previous study, statin therapy significantly increased lipoprotein(a) levels by 10–20% [19]. However, alirocumab was shown to be effective in reducing lipoprotein(a) levels and cardiovascular risk after a recent acute coronary syndrome [15]. Moreover, evolocumab inhibited the transient increase of lipoprotein(a) levels after AMI by inhibiting mature PCSK9, which binds to lipoprotein(a) [20]. In the current study, lipoprotein(a) levels increased in the control group but not in the evolocumab group. Thus, evolocumab may partly mitigate the increase in lipoprotein(a) levels by statins, suggesting its benefit for managing residual risk in patients with high lipoprotein(a) levels after AMI. Moreover, reports have shown that evolocumab specifically reduced lipoprotein(a) levels in patients with higher baseline lipoprotein(a) levels and tended to derive a greater coronary benefit from PCSK9 inhibition [21,22]. Additionally, higher levels of lipoprotein(a) are associated with an increased risk of cardiovascular events, irrespective of LDL levels [22]. The association between serum lipoprotein(a) levels and progression of atherosclerosis has also been reported [23]. In our study, baseline lipoprotein(a) levels were generally low, but evolocumab reduced lipoprotein(a) levels in the lipoprotein(a) > 20 mg/dL group. Our study suggests that these trends may be observed in the earlier phase of AMI.

### Limitations

The study has several limitations. First, this was a single-center, prospective, open-labeled randomized controlled trial, and the evaluators of all outcomes were blinded to the treatment allocation. Thus, we cannot rule out the influence of this design on the results. Second, the sample size of this study was small. Although the principal study found a significant change in LDL-C levels even with this sample size, the small sample size may cause a lower-powered statistical analysis on the conclusions drawn from the sub-analysis. Third, this study cannot evaluate the effect of evolocumab by itself without statin, although a previous study showed that evolocumab reduced lipoprotein(a) levels in patients resistant or intolerant to statin and/or fibrate therapy [24]. Finally, the study population comprised only Japanese patients; therefore, this research needs to be performed on a more diverse population in order to generalize the results.

## 4. Materials and Methods

### 4.1. Study Design and Participants

The design and main results of this study have been described previously (14). This sub-analysis was performed using data obtained from the previous study. Our study was a single-center, prospective, open-label, randomized controlled trial and included patients who experienced AMI and underwent primary PCI. The study was approved by the Ethics Committee of Kagawa Prefectural Central Hospital. All participants provided written informed consent. The investigation conformed to the principles outlined in the Declaration of Helsinki. This trial was registered at the University Hospital Medical Information Network Center (UMIN000028729). The data for this study are available from the corresponding author upon reasonable request.

The study design is detailed in the Supplementary Materials. Briefly, patients hospitalized for AMI (ST-elevation myocardial infarction (STEMI) or non-STEMI) within 24 h of symptom onset were eligible for the study. AMI was diagnosed according to the third universal definition of myocardial infarction [25]. Patients who fulfilled all inclusion and exclusion criteria were randomly assigned in a 1:1 ratio to the evolocumab and control groups after PCI. Randomization was performed using a computer-generated random sequence web response system and stratified by the center using random permuted blocks.

Within 24 h of the index PCI, patients were started on pitavastatin (2 mg/day) and subcutaneous evolocumab (140 mg). Evolocumab was administered every 2 weeks. In the control group, pitavastatin (2 mg/day) was started within 24 h of the PCI without evolocumab injection. The clinical follow-up visit was scheduled at 4 weeks.

### 4.2. PCI Procedure

PCI was performed using conventional techniques via the femoral or radial approach [26]. Patients received optimal medical therapy, such as dual antiplatelet agents, angiotensin-converting-enzyme inhibitors, β-blockers, and statins, for 1 year after PCI based on the guidelines for the secondary prevention of myocardial infarction from the Japanese Cardiology Society [27].

### 4.3. Outcomes

The main outcomes of this sub-analysis were changes in lipoprotein(a) levels from the baseline to 4 weeks. Lipoprotein(a) levels were divided into three groups with reference to the baseline levels, and each group was evaluated. Changes in subgroups were also evaluated. The parameters were evaluated for absolute change, percentage change, or both, from the baseline to 4 weeks.

### 4.4. Statistical Analysis

Continuous variables are expressed as mean ± standard deviation, and categorical variables are expressed as numbers (proportion). Comparisons of continuous variables in the baseline characteristics were analyzed using Student’s *t*-tests. Categorical data were compared using chi-squared analysis and Fisher’s test based on the sample size. Efficacy analysis was performed according to the treatment received based on an intention-to-treat analysis. We estimated group differences in the mean percentage change in lipoprotein(a) levels from baseline to 4 weeks and the interaction between the follow-up period and groups using mixed-effect linear regression models. The effects of evolocumab vs. control on lipoprotein(a) levels after 4 weeks were assessed in several subgroups defined by sex, age (<65 years, ≥65 years), baseline body mass index (<23 kg/m^2^, ≥23 kg/m^2^), prior PCI (yes, no), diabetes (yes, no), hypertension (yes, no), baseline estimated glomerular filtration rate (eGFR ≥ 60 mL/min/1.73 m^2^, <60 mL/min/1.73 m^2^), prior smoking (yes, no), current smoking (yes, no), prior statin therapy (yes, no), and baseline LDL-C (<120 mg/dL, ≥120 mg/dL). All comparisons and analyses were two-sided, with *p*-values < 0.05 considered statistically significant. All statistical analyses were performed using IBM SPSS Statistics 24 (IBM, Armonk, NY, USA).

## 5. Conclusions

In patients who underwent primary PCI for AMI, early initiation of evolocumab therapy in addition to a statin rapidly suppressed the increase in lipoprotein(a) levels compared with the use of statin alone. These effects were observed regardless of the baseline lipoprotein(a) levels and baseline characteristics. Early administration of a PCSK9 inhibitor plus a statin seems to be a feasible therapeutic approach for patients with AMI who undergo primary PCI, and it could decrease future cardiovascular events.

## Figures and Tables

**Table 1:** Baseline characteristics.

Characteristics	Evolocumab (*n* = 52)	Control (*n* = 50)	*p*-Value
Age (years)	66.4 ± 13.1	63.4 ± 14.0	0.34
Male sex	43 (82)	47 (94)	0.12
Body mass index, kg/m^2^	24.4 ± 4.4	25.2 ± 4.5	0.35
Diabetes mellitus	17 (32)	14 (28)	0.66
Hypertension	31 (59)	33 (66)	0.54
Smoker	25 (48)	18 (36)	0.23
Previous myocardial infarction	1 (1.9)	5 (10)	0.10
Previous PCI	4 (7.6)	10 (20)	0.08
Previous CABG	0	0	1.00
Peripheral artery disease	1 (1.9)	0	1.00
Stroke	0	3 (6.0)	0.11
Atrial fibrillation	3 (5.7)	3 (6.0)	1.00
eGFR, mL^−1^min^−1^ 1.73 m^2^	69.1 ± 25.1	70.0 ± 22.3	0.92
Statin treatment at baseline			0.82
Any statin	14 (26)	12 (24)	
No statin	38 (73)	38 (76)	
Index AMI event			0.14
STEMI	48 (92)	41 (82)	
NSTEMI	4 (7.6)	9 (18)	
Initial TIMI flow grade			0.51
Grade 0	39 (75)	35 (70)	
Grade 1	5 (9.6)	5 (10)	
Grade 2	5 (9.6)	7 (14)	
Grade 3	3 (5.7)	3 (6)	
Final TIMI flow grade			1.00
Grade 2	1 (2)	0 (0)	
Grade 3	51 (98)	50(100)	
Access site for PCI			0.73
Transfemoral approach	4 (7.6)	5 (10)	
Transradial approach	48 (92)	45 (90)	
Target coronary artery			0.47
Left anterior descending	25 (48)	23 (46)	
Left circumflex	7 (13)	5 (10)	
Right	20 (38)	22 (44)	

Values are presented as mean ± standard deviation or *n* (%). AMI, acute myocardial infarction; CABG, coronary artery bypass grafting; eGFR, estimated glomerular filtration rate; NSTEMI, non-ST-segment elevation myocardial infarction; PCI, percutaneous coronary intervention; STEMI, ST-segment elevation myocardial infarction; TIMI, thrombolysis in myocardial infarction.

**Table 2:** Changes in lipoprotein(a): baseline to 4 weeks.

Lipoprotein(a)	Evolocumab	Control	Mean Difference	*p*-Value
			(95% CI) *	
Baseline, mg/dL	14.9 ± 15.5	14.2 ± 15.1	0.8 (−6.3 to 8.0)	0.82
At week 4, mg/dL	14.2 ± 15.4	21.8 ± 20.7	−7.6 (−16.2 to 0.9)	0.08
Absolute change from baseline, mg/dL	−0.6 ± 6.6	7.5 ± 8.9	−8.4 (−12.2 to −4.7)	<0.001
% change from baseline, %	−2.7 ± 48.6	82.0 ± 135.9	−86.3 (−134.5 to −38.0)	0.01
Lipoprotein(a) ≤ 10	(*n* = 24)	(*n* = 29)		
Baseline, mg/dL	5.4 ± 3.0	5.7 ± 2.9	−0.3 (−1.9 to 1.3)	0.719
At week 4, mg/dL	5.5 ± 4.7	10.0 ± 7.1	−4.5 (−7.8 to −1.2)	0.008
Absolute change from baseline, mg/dL	0.04 ± 3.6	4.2 ± 6.3	−4.2 (−7.0 to −1.4)	0.004
% change from baseline, %	−1.2 ± 55.5	100.2 ± 167.5	−101.4 (−168.6 to −34.2)	0.004
10 < lipoprotein(a) ≤ 20	(*n* = 18)	(*n* = 12)		
Baseline, mg/dL	15.1 ± 2.9	15.0 ± 2.9	0.1 (−2.0 to 2.4)	0.88
At week 4, mg/dL	15.6 ± 8.5	26.3 ± 13.7	−10.6 (−20.0 to −1.2)	0.02
Absolute change from baseline, mg/dL	0.5 ± 6.9	11.3 ± 12.2	−10.8 (−19.0 to −2.5)	0.013
% change from baseline, %	−0.4 ± 46.9	71.1 ± 78.7	−71.5 (−125.1 to −18.0)	0.012
Lipoprotein (a) > 20	(*n* = 7)	(*n* = 8)		
Baseline, mg/dL	46.7 ± 18.3	43.8 ± 15.1	2.8 (−16.3 to 21.9)	0.752
At week 4, mg/dL	40.8 ± 21.6	57.7 ± 18.8	−16.8 (−39.8 to 6.1)	0.136
Absolute change from baseline, mg/dL	−5.8 ± 11.2	13.8 ± 6.2	−19.7 (−30.5 to −8.8)	0.003
% change from baseline, %	−14.0 ± 25.5	32.2 ± 15.7	−46.3 (−71.2 to −21.3)	0.002

Values are presented as mean ± standard deviation. The mean difference was calculated with a linear mixed-effects model adjusting for statin treatment at baseline. * Evolocumab minus placebo. CI, confidence interval.

**Table 3:** Changes in lipoprotein(a) levels in patients with and without diabetes.

Lipoprotein(a)	Evolocumab	Control	Mean Difference	*p*-Value
			(95% CI) *	
Patients with diabetes				
*n*	16	14		
Baseline, mg/dL	17.3 ± 20.9	10.2 ± 8.6	7.1 (−4.3 to 19.0)	0.22
At week 4, mg/dL	15.5 ± 19.8	20.0 ± 16.1	−4.4 (−17.9 to 9.0)	0.50
Absolute change from baseline, mg/dL	−1.8 ± 6.1	9.7 ± 12.3	−11.5 (−19.2 to −3.9)	0.005
% Change from baseline, %	−15.8 ± 45.5	168.2 ± 222.1	−184.1 (−313.8 to −54.4)	0.009
Patients without diabetes				
*n*	35	35		
Baseline, mg/dL	13.2 ± 12.1	15.8 ± 16.9	−2.6 (−9.6 to 4.4)	0.46
At week 4, mg/dL	13.4 ± 12.6	22.5 ± 22.4	−9.0 (−17.8 to −0.3)	0.04
Absolute change from baseline, mg/dL	0.2 ± 6.7	6.6 ± 7.2	−6.4 (−9.8 to −3.1)	<0.001
% Change from baseline, %	8.5 ± 52.8	47.5 ± 54.1	−38.9 (−64.4 to −13.4)	0.003

Values are presented as mean ± standard deviation. The mean difference was calculated with a linear mixed-effects model adjusting for statin treatment at baseline. * Evolocumab minus placebo. CI, confidence interval.

## Data Availability

The data for this study are available from the corresponding author upon reasonable request.

## Supplementary Materials

The following supporting information can be downloaded at: https://www.mdpi.com/article/10.3390/jcdd9050153/s1, Supplemental Methods: Inclusion and exclusion criteria, Study design; Table S1: Change in lipid levels.
