# Supplementary material for: Effect of Early Initiation of Evolocumab on Lipoprotein(a) in Patients with Acute Myocardial Infarction: Sub-Analysis of a Randomized Controlled Trial
Source: J Cardiovasc Dev Dis. 2022 May 12;9(5):153. doi: 10.3390/jcdd9050153 (PMC9144976; doi:10.3390/jcdd9050153)

Supplemental Methods

1. Inclusion and exclusion criteria

Inclusion criteria

- AMI* patients undergoing successful primary PCI within 24 hours after onset of symptoms
- Male or female individual aged ≥ 20 years and ≤ 90 years
- LDL-C ≥ 70mg/dL at screening

Exclusion criteria

- Cardiogenic shock after PCI
- Post-resuscitation
- Chronic dialysis
- Severe liver disease
- Active malignancy
- Inability to undergo statin therapy
- Undergoing therapy involving a PCSK9 inhibitor
- History of side effects of statins and PCSK9 inhibitors
- Pregnancy or possibility of pregnancy

*Definition of myocardial infarction (AMI)

The term MI should be used when there is evidence of myocardial necrosis in a clinical setting consistent with myocardial ischemia. Under these conditions, any one of the following criteria (A and B) meets the diagnosis for myocardial infarction. [1]

A. Spontaneous MI (type 1)

To identify a type 1 MI, patients should demonstrate spontaneous symptoms of myocardial ischemia unprovoked by supply/demand inequity, together with ≥1 of the following criteria:

- Cardiac biomarker elevation: Troponin is the preferred marker for adjudicating the presence of acute MI. At least one value should show a rise and/or fall from the lowest cut-point providing 10% imprecision (typically the upper reference limit for the troponin run per standard of clinical care). Creatine kinase-MB is a secondary choice of marker to troponin; a rise in CK-MB above the local upper reference limit would be consistent with myocardial

injury.

- ECG changes consistent with new ischemic changes

– ECG changes indicative of new ischemia (new ST-T changes or new left bundle branch block [LBBB]) or ECG manifestations of acute myocardial ischemia (in the absence of left ventricular hypertrophy [LVH] and LBBB):

– Development of pathological Q waves in the ECG

1. Any Q-wave in leads V2–V3 ≥0.02 seconds or QS complex in leads V2 and V3
2. Q-wave ≥0.03 seconds and ≥0.1 mV deep or QS complex in leads I, II, aVL, aVF, or V4-V6 in any two leads of a contiguous lead grouping (I, aVL, V6; V4-V6; II, III, and aVF)

– ST elevation: New ST elevation at the J-point in two contiguous leads with the cut-off points: ≥0.2 mV in men or ≥0.15 mV in women in leads V2–V3 and/or ≥0.1 mV in other leads

– ST depression and T-wave changes: New horizontal or down-sloping ST depression ≥0.05 mV in two contiguous leads and/or T inversion ≥0.1 mV in two contiguous leads with prominent R-wave or R/S ratio >1

- Imaging evidence of new non-viable myocardium or new wall motion abnormality

B. “Demand”-related (type 2) MI

Patients with type 2 MI should be considered under similar diagnostic criteria as a type 1 MI; however, type 2 MI should be considered present when myocardial ischemia and infarction are consequent to supply/demand inequity, rather than a spontaneous plaque rupture and coronary thrombosis.

C. Percutaneous coronary intervention (PCI)-related MI (type 4a/4b)

For PCI in patients with normal baseline troponin values, elevations of cardiac biomarkers above the 99th percentile URL within 24 hours of the procedure are indicative of peri-procedural myocardial necrosis. By convention, increases of biomarkers >3 × 99th percentile URL (troponin or CK-MB >3 × 99th percentile URL) are consistent with PCI-related MI.

Where the cardiac biomarker is elevated prior to PCI, a ≥20% increase in the value of the second cardiac biomarker sample within 24 hours of PCI and documentation that cardiac biomarker values were decreasing (two samples ≥6 hours apart) prior to the suspected recurrent MI are

consistent with PCI-related MI.
Symptoms of cardiac ischemia are not required.

D. Coronary artery bypass grafting (CABG)-related MI (type 5)
For CABG in patients with normal baseline troponin values, elevation of cardiac biomarkers above the 99th percentile URL within 72 hours of the procedure is indicative of peri-procedural myocardial necrosis. By convention, an increase of biomarkers >5 × 99th percentile URL (troponin or CK-MB >5 × 99th percentile URL) plus at least one of the following is consistent with CABG-related MI:

- New pathological Q waves in at least two contiguous leads on the ECG that persist for 30 days, or new LBBB
- Angiographically documented new graft or native coronary artery occlusion
- Imaging evidence of new loss of viable myocardium

If the cardiac biomarker is elevated prior to CABG, a ≥20% increase in the value of the second cardiac biomarker sample within 72 hours of CABG and documentation that cardiac biomarker values were decreasing (two samples ≥6 hours apart) prior to the suspected recurrent MI plus new pathological Q-waves in ≥2 contiguous leads on the electrocardiogram; or new LBBB, angiographically documented new graft, or native coronary artery occlusion; or imaging evidence of new loss of viable myocardium are consistent with a periprocedural MI after CABG. Symptoms of cardiac ischemia are not required.

Reference

1.Thygesen K, Alpert JS, Jaffe AS, et al. Third universal definition of myocardial infarction. J Am Coll Cardiol. 2012;60(16):1581-1598.

2. Study design

Patients who successfully underwent primary PCI within 24 hours after onset of AMI were randomly assigned 1:1 to receive evolocumab 140 mg (subcutaneously injected within 24 hours after PCI and then at every 2 weeks) or control.

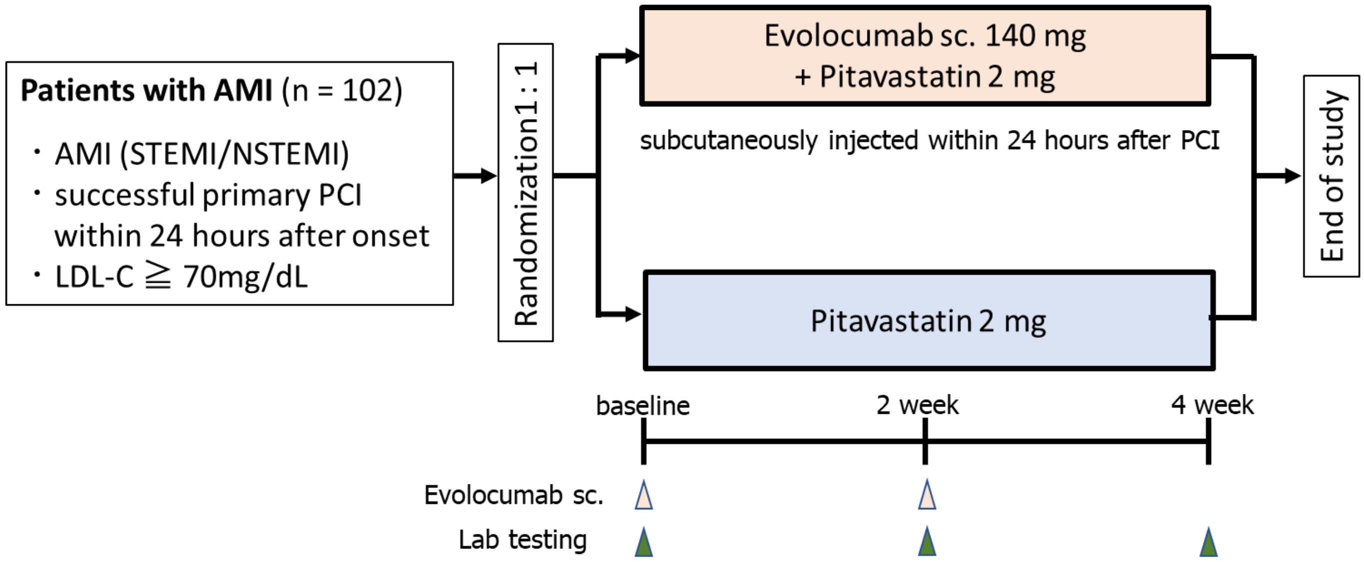

Supplement: Supplementary file 1 [file jcdd-09-00153-s001.zip › Supplement method.pdf]
