# Supplementary material for: Effect of Early Initiation of Evolocumab on Lipoprotein(a) in Patients with Acute Myocardial Infarction: Sub-Analysis of a Randomized Controlled Trial
Source: J Cardiovasc Dev Dis. 2022 May 12;9(5):153. doi: 10.3390/jcdd9050153 (PMC9144976; doi:10.3390/jcdd9050153)
Supplement: Supplementary file 1 [file jcdd-09-00153-s001.zip › Table S1.pdf]

Table S1. Changes in Lipid Levels

Lipid levels	Evolocumab	Control	Mean difference (95% CI)	p value
Total cholesterol at baseline, mg/dL	187.9 ± 40.6	188.3 ± 38.5	-1.1 (-19.5 to 17.1)	0.90
Total cholesterol at week 4, mg/dL	91.2 ± 19.8	145.1 ± 28.8	-50.1 (-61.6 to -38.6)	<0.001
Absolute change from baseline, mg/dL	-96.7 ± 39.8	-43.2 ± 39.2	-51.3 (-69.2 to -33.4)	<0.001
% change from baseline, %	-50.0 ± 12.4	-20.9 ± 18.2	-28.6 (-35.7 to -21.6)	<0.001
LDL at baseline, mg/dL	120.8 ± 30.6	124.7 ± 33.6	-0.2 (-14.8 to 14.3)	0.972
LDL at week 4, mg/dL	27.3 ± 13.4	79.8 ± 23.2	-48.6 (-57.5 to -39.8)	<0.001
Absolute change from baseline, mg/dL	-93.4 ± 32.6	-44.8 ± 32.1	-48.9 (-63.7 to -34.1)	<0.001
% change from baseline	-76.1 ± 12.7	-33.1 ± 22.0	-43.9 (-52.1 to -35.6)	<0.001
Triglyceride at baseline, mg/dL	104.8 ± 101.0	110.2 ± 83.9	-15.7 (-59.3 to 27.9)	0.48
Triglyceride at week 4, mg/dL	116.6 ± 52.7	131.1 ± 61.2	-8.4 (-35.3 to 18.3)	0.53
Absolute change from baseline, mg/dL	11.8 ± 92.5	21.1 ± 94.7	7.2 (-36.5 to 50.9)	0.74
% change from baseline, %	75.0 ± 123.8	76.7 ± 115.7	21.0 (-34.8 to 76.8)	0.46
HDL-C at baseline, mg/dL	44.7 ± 12.2	43.2 ± 9.2	0.6 (-4.3 to 5.7)	0.79
HDL-C at week 4, mg/dL	44.8 ± 11.5	42.2 ± 9.7	1.8 (-2.9 to 6.7)	0.44
Absolute change from baseline, mg/dL	0.04 ± 9.4	-1.02 ± 8.3	1.2 (-2.9 to 5.4)	0.57

% change from baseline, %	2.8 ± 21.4	-0.7 ± 20.3	3.4 (-6.3 to 13.3)	0.48
Non-HDL at baseline, mg/dL	143.1 ± 37.8	145.1 ± 36.7	-0.5 (-16.4 to 17.4)	0.95
Non-HDL at week 4, mg/dL	46.3 ± 14.7	102.9 ± 25.4	-52.0 (-61.7 to -42.4)	<0.001
Absolute change from baseline, mg/dL	-96.7 ± 38.2	-42.2 ± 37.0	-52.5 (-69.6 to -35.4)	<0.001
% change from baseline, %	-66.2 ± 12.5	-26.0 ± 22.1	-39.3 (-48.0 to -31.5)	<0.001
Apoprotein B at baseline, mg/dL	95.1 ± 22.9	97.2 ± 21.9	-0.8 (-11.1 to 9.5)	0.88
Apoprotein B at week 4, mg/dL	34.1 ± 12.0	75.2 ± 15.8	-38.4 (-45.0 to -31.9)	<0.001
Absolute change from baseline, mg/dL	-60.9 ± 23.2	-21.9 ± 22.0	-37.6 (-47.9 to -27.3)	<0.001
% change from baseline, %	-62.8 ± 13.7	-19.8 ± 20.6	-42.7 (-50.7 to -34.7)	<0.001
Apoprotein C3 at baseline, mg/dL	8.8 ± 5.5	8.6 ± 3.6	-0.4 (-2.6 to 1.7)	0.7
Apoprotein C3 at week 4, mg/dL	7.2 ± 2.1	8.4 ± 2.8	-1.0 (-2.2 to 0.1)	0.07
Absolute change from baseline, mg/dL	-1.6 ± 4.9	-0.2 ± 3.7	-0.6 (-2.7 to 1.3)	0.52
% change from baseline, %	-8.7 ± 25.7	4.4 ± 35.6	-8.8 (-23.3 to 5.6)	0.23
Apoprotein A1 at baseline, mg/dL	122.0 ± 25.0	121.4 ± 18.9	2.0 (-8.2 to 12.2)	0.70
Apoprotein A1 at week 4, mg/dL	129.8 ± 22.7	121.0 ± 23.4	8.3 (-2.1 to 18.8)	0.12
Absolute change from baseline, mg/dL	7.7 ± 22.0	-0.3 ± 17.6	10.3 (1.0 to 19.7)	0.03
% change from baseline, %	8.4 ± 18.9	0.2 ± 15.1	10.0 (1.9 to 18.0)	0.02

Small dense LDL at baseline, mg/dL	34.7 ± 20.1	36.7 ± 19.3	-2.3 (-11.5 to 6.8)	0.61
Small dense LDL at week 4, mg/dL	9.4 ± 4.3	23.0 ± 10.4	-12.6 (-16.3 to -8.8)	<0.001
Absolute change from baseline, mg/dL	-25.3 ± 18.7	-13.7 ± 19.6	-10.2 (-19.1 to -1.2)	0.03
% change from baseline, %	-67.3 ± 17.2	-13.8 ± 109.7	-48.3 (-85.4 to -11.3)	0.01

Values have been given as mean ± standard deviation; the mean difference was calculated with a linear mixed-effects model adjusting for statin treatment at baseline.

CI, confidence interval; HDL, high-density lipoprotein cholesterol; LDL, low-density lipoprotein cholesterol.
